# Impaired Synaptic Activity in the Basolateral Amygdala Is Associated With an Alcohol Use Disorder‐Like Vulnerable Phenotype in Male Rats

**DOI:** 10.1111/adb.70091

**Published:** 2025-10-14

**Authors:** Davide Cadeddu, Erika Lucente, Mia Ericson, Bo Söderpalm, Louise Adermark, Ana Domi

**Affiliations:** ^1^ Department of Pharmacology, Institute of Neuroscience and Physiology, The Sahlgrenska Academy University of Gothenburg Gothenburg Sweden; ^2^ Addiction Biology Unit, Department of Psychiatry and Neurochemistry, Institute of Neuroscience and Physiology, The Sahlgrenska Academy University of Gothenburg Gothenburg Sweden

**Keywords:** addiction, alcohol, amygdala, BLA, electrophysiology, ethanol, self‐administration

## Abstract

Alcohol use disorder (AUD) is associated with a loss of control over alcohol use, putatively driven by maladaptive changes in neural circuitries, including the basolateral amygdala (BLA). The BLA, known for its role in emotional regulation and associative learning, contributes to the reinforcement of alcohol‐related behaviours, making it a critical target for understanding the underlying mechanisms of vulnerability to AUD. To further outline the role of BLA neurotransmission in AUD, we combined a multisymptomatic 0/3 criteria rodent model with electrophysiological whole‐cell recordings to identify the association between neurophysiological parameters in the BLA and vulnerability to AUD‐like progression. Our results demonstrate that when assessed after 4 months of voluntary alcohol consumption, rats can be subcategorized as resilient or vulnerable to AUD‐like behaviour. Electrophysiological recordings, performed directly after alcohol self‐administration, demonstrated that rats manifesting an AUD‐like vulnerable phenotype presented a reduced frequency and amplitude of spontaneous excitatory post‐synaptic currents (sEPSCs), indicating suppressed activation via glutamatergic inputs. Disinhibition induced by GABAA receptor antagonist did not differ between groups, and field potential recordings demonstrated reduced stimulus/response curves further supporting a hypoglutamatergic state. Additionally, the intrinsic excitability of BLA neurons was selectively decreased in vulnerable rats compared to both resilient and water control rats. Importantly, addiction score correlated with both synaptic transmission and intrinsic excitability of BLA neurons. Overall, our findings suggest that hypoexcitability of BLA neurons may represent a neurobiological underpinning that contributes to the development and persistence of alcohol addiction‐like behaviours following protracted alcohol exposure.

## Graphical abstract

Following long‐term operant alcohol self‐administration, Wistar rats were categorised based on their propensity to express an AUD‐like behavioural phenotype. Electrophysiological recordings, conducted shortly after alcohol intake, demonstrated a hypoglutamatergic state and reduced intrinsic excitability in the basolateral amygdala selectively in vulnerable rats. Moreover, neurotransmission correlated with both addiction score and alcohol intake.

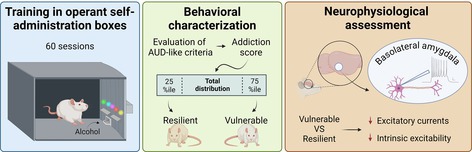

## 1 Introduction

Although alcohol is commonly used for recreational purposes, a significant minority develop alcohol use disorder (AUD), a medical condition that poses a substantial burden on individuals, society and healthcare systems [1, 2]. The disease progression follows a trajectory marked by disruptions in synaptic function and neural circuit activity, progressively leading to uncontrolled drinking episodes in vulnerable individuals [3, 4]. A deeper understanding of alcohol‐induced alterations in molecular and synaptic mechanisms driving individual vulnerability to AUD is essential for developing therapies that can reverse neural plasticity and the associated maladaptive behaviours.

Our recent work addresses this need by demonstrating that individual vulnerability to AUD‐like behaviour is associated with unique changes in excitatory synaptic transmission in the rats' prelimbic cortex (PL) that are absent in animals maintaining controlled alcohol intake over time [5]. Specifically, we observed a reduction in PL presynaptic activity that may reflect alterations in the excitatory drive from subcortical brain regions representing a circuitry that possibly sustains AUD behaviour. A key input region that projects to the PL is the basolateral amygdala (BLA) [6]. Part of the amygdaloid complex, the BLA is reciprocally connected to the PL and plays a central role in modulating reward learning and instrumental reward‐seeking behaviours [7, 8]. On the neuronal level, the BLA is a cortical‐like structure composed of approximately 80% glutamatergic projection neurons and 20% inhibitory GABAergic interneurons [9]. This balance between excitatory and inhibitory neurons regulates the synaptic activity within the BLA and modulates its output to other brain regions.

Given its primary role in controlling negative emotional states that can trigger relapse in AUD patients, the majority of the animal research has focused on BLA neuronal activity in alcohol‐seeking behaviours under reinstatement conditions. These studies highlight BLA glutamatergic signalling as a critical driver in the reinstatement of cue‐evoked alcohol‐seeking behaviours [10, 11, 12]. Although they suggest that increased BLA glutamate transmission may trigger alcohol‐seeking behaviour in relapse models, little is known about the specific role of BLA excitatory transmission when drug seeking becomes ‘compulsive’ under conditions marked by multiple signs of loss of control over alcohol use.

In this context, we hypothesized that alterations in BLA synaptic excitatory transmission contribute to the compulsive‐like nature of alcohol‐seeking behaviour in rats exhibiting an addiction‐like phenotype. Following a prolonged period of alcohol self‐administration, we measured multiple signs of loss of control over alcohol use, including (i) inability to cease alcohol seeking, (ii) higher motivation for alcohol and (iii) continued alcohol use despite adverse consequences. We then investigated the changes in synaptic glutamatergic transmission through whole‐cell patch‐clamp recordings in BLA neurons, comparing rats displaying AUD‐like behaviours with those exhibiting resilience maintaining controlled alcohol consumption.

## 2 Methods and Materials

### 2.1 Subjects

**FIGURE 1:**
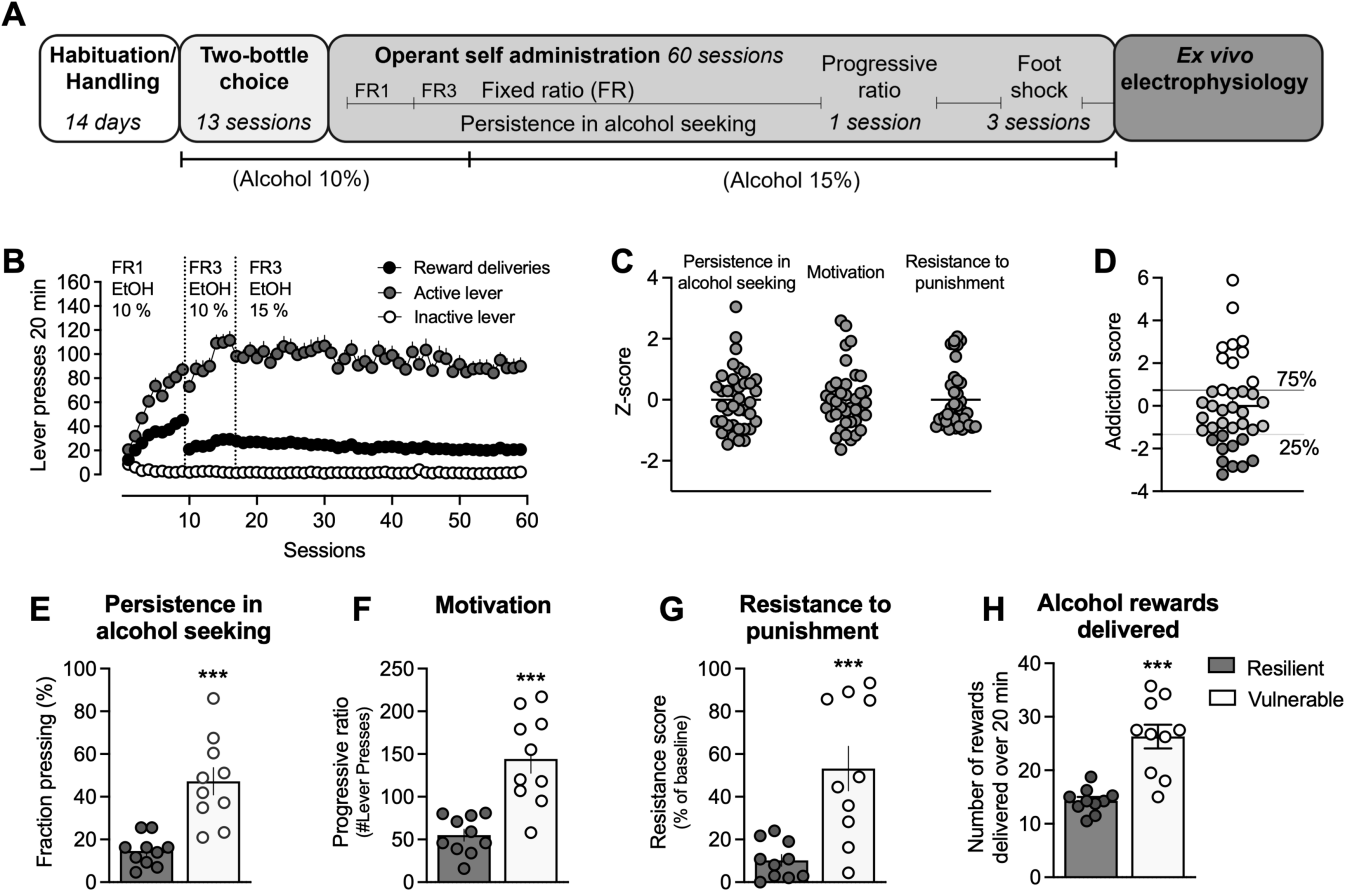
Addiction‐like phenotypic traits in outbred Wistar rats. (A) Timeline for the experiments. (B) Time‐course graph demonstrating active and inactive lever presses together with the delivery of alcohol rewards for all rats during operant alcohol self‐administration. (C) Normalized z‐scores were calculated for each rat to evaluate persistence in responding for alcohol, motivation for the drug and resistance to punishment when alcohol delivery was paired with a footshock. (D) The Global Addiction Score (GAS) was obtained as the sum of the z‐scores for each criterion for each rat. Individuals that scored above the 75th percentile were considered vulnerable, whereas those below the 25th percentile were considered resilient. (E–G) Compared to resilient rats, vulnerable rats showed increased response for alcohol‐seeking during drug‐free periods (E), higher motivation to consume alcohol under increased effort (F) and greater resistance to footshock punishment during operant responding for alcohol (G). (H) The number of alcohol rewards earned differed significantly between resilient and vulnerable rats. Values are presented as mean (±SEM). Each dot in (C–H) represents one rat.

Male Wistar rats *n* = 56 (Charles River, Sulzfeld, Germany), weighing 230–250 g (around 8–9 weeks old) upon arrival at the facility, were group‐housed in pairs in a temperature and humidity‐controlled environment under a 12‐h reversed light/dark cycle (lights on at 7:00 pm, lights off at 7:00 am). Rats had free access to food and water *ad libitum*. Animals were given a 1‐week acclimation period at the facility prior to the start of the experiments. Eight rats were kept in their home cages during the full experiment and used as water drinking controls. In addition, another eight drug‐naïve rats were used for validating that recorded currents were glutamatergic. Procedures were conducted in accordance with the National Committee for Animal Research in Sweden and approved by the Local Ethics Committee for Animal Care and Use at Gothenburg University. A timeline for the study is presented in Figure 1A.

### 2.2 Alcohol Self‐Administration

Alcohol self‐administration (SA) training was conducted as previously described [5, 13]. Prior to operant responding training, rats (*n* = 40) underwent a 4‐week intermittent two‐bottle choice alcohol procedure (24 h, 3 times a week, 10% alcohol) to facilitate operant responding acquisition and avoid sucrose fading or water deprivation. Rats were then trained to self‐administer alcohol (10% on a fixed‐ratio 1 [FR1]) schedule of reinforcement followed by FR3 and alcohol 15% that was maintained for the rest of the training (over 60 SA sessions). Each session lasted 30 min and consisted of two 10‐min drug periods alternated by one 10‐min period without access to alcohol. Pressing the active lever during the drug phase delivered 0.1 mL of ethanol, illuminated a cue light above the lever for 5 s and initiated a 10‐s timeout. Responses on the inactive lever were also recorded but had no programmed outcome. The 10‐min period when alcohol was not available was indicated by the activation of the house light. During this period, pressing the active lever did not result in any scheduled consequence. Following each session, the experimenter verified that no alcohol remained in the self‐administration box receptacle.

### 2.3 Evaluation of the Addiction‐Like Behaviours

Rats were categorized as resilient or vulnerable to develop AUD‐like behaviour based on their individual z‐score when assessing three distinct behaviours: (i) persistence in alcohol‐seeking, (ii) motivation for alcohol and (iii) resistance to punishment to receive an alcohol reward, as previously described [5]. (i) Persistence in alcohol‐seeking was measured daily as ‘fraction pressing’ during the 10‐min no‐drug periods. Each rat's daily lever pressing was classified as ‘pressing’ [1] or ‘non‐pressing’ (0) based on whether it exceeded the 66th percentile of the population's distribution, averaged over 5 days to avoid fluctuations. ‘Fraction pressing’, expressed in percentage, was the algebraical sum of the ‘pressing days’ relative to total days for each rat and combined into a z‐score based on the group mean. (ii) Motivation for alcohol was measured by a progressive ratio (PR) schedule of reinforcement. The ratio of responses per alcohol infusion was 1, 2, 3, 4, 6, 8, 10 and 12, after which the ratio increased in steps of 4. The breakpoint was defined as the final ratio completed, with sessions ending after 30 min had passed since the last reinforced response. For each rat, a z‐score was calculated based on the (iii) resistance to punishment, which was assessed over three consecutive 10‐min sessions, with the resistance score (Rs) calculated by comparing average rewards earned in three baseline sessions to those in three punished sessions. In a FR3, after the first lever press, the green cue light was illuminated to signal the presence of the footshock, the second lever press resulted in a footshock (0.25 mA, 0.5 s), and the third lever press resulted in both the delivery of an alcohol reward and a 5‐s illumination of the cue light above the lever. If animals failed to complete the FR3 within 1 min, the green light turned off, and the sequence restarted.

The addiction score (AS) was calculated as the algebraic sum of standardized scores (z‐scores) of each of the three addiction‐like behaviours. Rats with an addiction score above the 75th percentile of the entire population were classified as vulnerable, whereas those below the 25th percentile were considered resilient [14, 15].

### 2.4 Electrophysiological Studies

#### 2.4.1 Brain Slice Preparation

Following re‐baselining, rats continued to gain access to the self‐administration boxes until electrophysiological recordings were conducted. To minimize the discrepancy in time between the first and last animal recorded, several animals were recorded each day, focusing on vulnerable and resilient rats. In parallel, ethanol‐naïve rats that had remained in their home cage during the experiment were used as a reference to assess the direction of putative neuroplasticity. On the day of the experiment, alcohol‐consuming rats were taken directly from the self‐administration box and brain slice preparation performed as previously reported [16]. In brief, rats were deeply anaesthetized, and the brain was rapidly removed and submerged in ice‐cold modified artificial cerebrospinal fluid (aCSF) containing (in mM) 220 sucrose, 2 KCl, 0.2 CaCl_2_, 6 MgCl_2_, 26 NaHCO_3_, 1.3 NaH_2_PO_4_ and 10 D‐glucose. Coronal brain slices (250 μm) containing the BLA were sectioned and transferred to aCSF containing (in mM) 124 NaCl, 4.5 KCl, 2 CaCl_2_, 1 MgCl_2_, 26 NaHCO_3_, 1.2 NaH_2_PO_4_ and 10 D‐glucose, continuously bubbled with a gas mixture of 95% O_2_/5% CO_2_. Before starting electrophysiological experiments, slices were incubated in aCSF for 30 min at 33°C and then kept at room temperature for the rest of the day.

#### 2.4.2 Whole‐Cell Recordings

Neurons located in the BLA were visualized under a Nikon Eclipse FN‐1 microscope equipped with a 10×/0.30 objective. Recording pipettes were prepared from borosilicate glass (2.5–5.5 MΩ, Sutter Instruments, Novato, CA) and filled with an internal solution containing (in mM) 135 K‐gluconate, 20 KCl, 2 MgCl_2_, 0.1 EGTA, 10 Hepes, 2 Mg‐ATP and 0.3 Na‐GTP. The pH was adjusted to 7.3 with KOH, and osmolarity was adjusted to 295 mOsm with sucrose. To record spontaneous excitatory post‐synaptic currents (sEPSCs), the membrane voltage was clamped at −65 mV using a MultiClamp 700‐B amplifier (Molecular Devices, Axon CNS, San Jose, CA), digitized at 10 kHz, and filtered at 2 kHz using Clampex (Molecular Devices). In a subset of the recorded neurons, a current‐clamp protocol was performed, where current was injected in 20 pA steps from −80 to 300 pA with a 1000 ms duration to hyperpolarize and depolarize the membrane. Recordings were conducted without GABA receptor antagonists in the aCSF. To validate that recorded sEPSCs derived from glutamatergic post‐synaptic currents, a subset of recordings was performed during bath perfusion of the competitive AMPA/kainate receptor antagonist 6‐cyano‐7‐nitroquinoxaline‐2,3‐dione (CNQX, 20 μM) and the NMDA receptor antagonist 2‐amino‐5‐phosphonovaleric acid (APV, 50 μM).

The neurons recorded were selected based on shape and size and assumed to be glutamatergic, but this was not further verified after recording. Thus, although the shape and parameters of both sEPSCs and action potentials were consistent with those typically observed in glutamatergic neurons, it is possible that a subfraction of recordings derives from non‐fast spiking GABAergic interneurons. Neurons displaying neurophysiological properties resembling fast spiking interneurons were excluded from the study. Only recordings with a stable series resistance that varied less than 20% and did not exceed 30 MΩ were included in the analysis.

#### 2.4.3 Field Potential Recordings

Field potentials were conducted to complement whole‐cell recordings and were performed in the BLA as previously described [17]. In brief, slices were perfused with a constant flow of preheated aCSF (30°C) and field excitatory post‐synaptic potentials (fEPSPs) were evoked at a frequency of 0.05 Hz with a stimulation electrode (World Precision Instruments, FL, USA; type TM33B) positioned locally, 0.2–0.3 mm from the recording electrode (borosilicate glass, 2.5–4.5 MΩ, World Precision Instruments). After recording a stable baseline for 10 min, the GABA_A_ receptor antagonist bicuculline (20 μM) was bath perfused for 25 min. To determine changes in evoked excitatory potentials, stimulus/response curves were recorded by stepwise increasing the stimulation intensity. In a subset of recordings, slices were bath perfused with a cocktail of CNQX (20 μM) and APV (50 μM) to validate that evoked potentials reflected glutamatergic signalling.

### 2.5 Statistics

Data are presented as mean ± standard error and were analysed by means of Student's *t*‐test comparison or one‐way or repeated‐measures analysis of variance (ANOVA) according to experimental design. Levene's test was applied to check for homogeneity of variance, and Mauchly's test assessed sphericity in repeated measures (RM)‐ANOVA. Significance was set at *p* < 0.05, and ANOVA was followed by Newman–Keuls post hoc test when appropriate. A Pearson's correlations were performed to examine the relationship between behavioural and electrophysiological measures. Data were analysed using STATISTICA, stat soft 13.0 (RRID:SCR_014213), Clampfit 10.2 (Molecular devices, Axon CNS, CA, United States) and Minianalysis 6.0 (Synaptosoft).

## 3 Results

### 3.1 Sub‐Dimensions of Addiction‐Like Behaviour in Outbred Wistar Rats

Rats (*n* = 40) acquired and maintained stable alcohol self‐administration levels over 60 daily sessions, 5 days a week (Figure 1A,B). Two rats were excluded from the study as they failed to acquire alcohol self‐administration. Based on their performance across the three AUD‐like criteria (Figure 1C), rats were separated based on their addiction score, and the top 25% were classified as vulnerable (*n* = 10), whereas the lower 25% were classified as resilient (*n* = 10) (Figure 1C,D).

When separated based on addiction score, the two groups represented opposite extremes in each of these behaviours. Rats that did not exhibit the characteristics of addiction‐like behaviours demonstrated resilience towards persistence in alcohol seeking when alcohol was not available (Figure 1E), a lack of motivation to exert increasing effort for a single alcohol infusion (Figure 1F) and avoidance of alcohol self‐administration during punishment (Figure 1G). Vulnerable rats, on the other hand, showed an inability to stop alcohol seeking during drug‐free periods (*t*_18_ = 4.823, *p* < 0.001) (Figure 1E), increased motivation to consume alcohol under increased effort (*t*_18_ = 4.934, *p* < 0.001) (Figure 1F) and greater resistance to footshock punishment during operant responding for alcohol (*t*_18_ = 3.994, *p* < 0.001) (Figure 1G). The propensity to develop AUD‐like behaviour was further associated with the number of rewards earned, demonstrating a significant difference in the number of reward deliveries between the two (Figure 1I). The receptacle was always empty when checked between the sessions, indicating that the ethanol rewards earned were consumed.

### 3.2 Altered Spontaneous Synaptic Activity in the BLA of Vulnerable Rats

**FIGURE 2:**
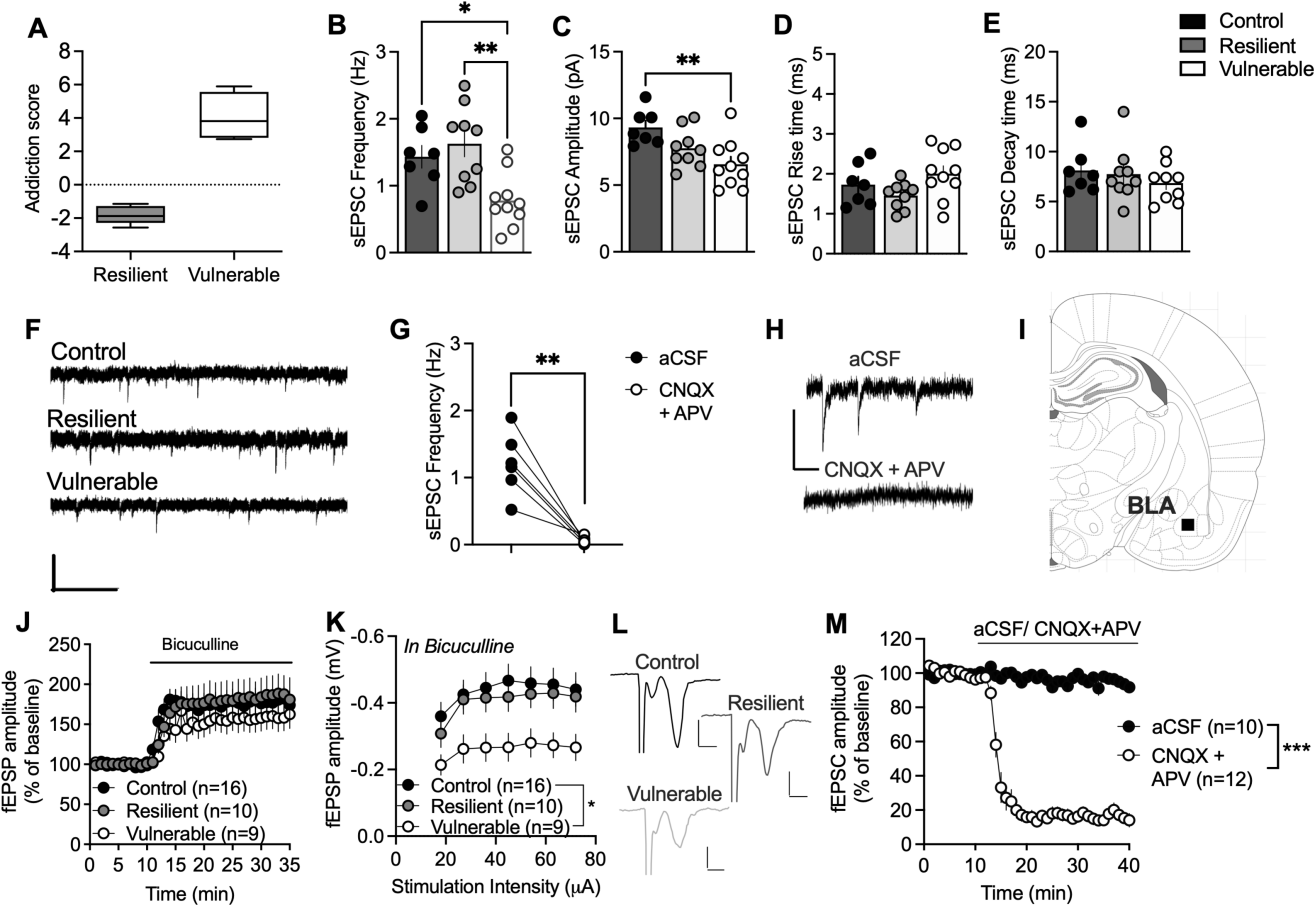
Addiction‐like phenotypic traits are associated with alterations in BLA spontaneous excitatory neurotransmission. (A) Addiction score differed significantly between the two groups. (B) Whole‐cell recordings performed in voltage‐clamp mode showed that the frequency of spontaneous events was significantly reduced in BLA neurons from vulnerable rats compared to neurons from resilient rats and water‐consuming controls. (C) The decrease in frequency was accompanied by a reduction in sEPSC amplitude in vulnerable rats compared to water controls. (D,E) Neither rise time nor decay time varied between the groups. (F) Representative traces showing measured sEPSCs for each recorded group. Calibration: 1 s, 40 pA. (G) Recorded currents were blocked by a cocktail of antagonists targeting AMPA and NMDA receptors, demonstrating that recordings reflect sEPSCs. (H) Example traces demonstrating recorded sEPSCs in aCSF and following 10‐min bath perfusion of CNQX (10 μM) and APV (50 μM). Calibration: 50 pA, 200 ms. (I) Schematic drawing marking the area of recording. (J) Field potential recordings demonstrated no significant group differences with respect to GABAergic tone on GABAA receptors. (K) Stimulus/response curves revealed a reduced amplitude of evoked fEPSP in brain slices from vulnerable rats, supporting a hypoglutamatergic state. (L) Example traces from field potential recordings demonstrating evoked fEPSPs. Calibration: 0.2 mV, 0.02 ms. (M) Bath perfusion of antagonists targeting AMPA and NMDA receptors inhibited evoked field potentials, demonstrating that recordings reflect glutamatergic neurotransmission. Values are presented as mean (±SEM). *n* = number of cells (C–F) or slices (H,I). Recordings are based on 4–8 animals/treatment group.

Ex vivo electrophysiological whole‐cell recordings were conducted to test the hypothesis that AUD‐like behaviour would be associated with distinct neurophysiological properties in the BLA. As a control, recordings were also performed in ethanol‐naïve rats, housed in parallel. Addiction score differed significantly between the two groups of rats selected for electrophysiological recordings (*t*_7_ = 8.354, *p* < 0.001) (Figure 2A). Recordings of spontaneous excitatory neurotransmission in voltage‐clamp mode highlighted a significant effect on sEPSC firing frequency as a function of AUD‐like behaviour when compared to water drinking control rats (*F*_2,22_ = 8.205, *p* = 0.0022) (Figure 2B). Rats that manifested AUD‐like traits presented reduced frequency of sEPSCs inputs onto BLA neurons compared to resilient (*p* < 0.01) and control groups (*p* < 0.05). The amplitude of sEPSCs also differed between groups (*F*_2,23_ = 6.593, *p* = 0.0055), with a significant decrease observed in vulnerable rats compared to controls (*p* = 0.0039), but not between vulnerable and resilient rats (*p* = 0.2290) (Figure 2C). Other post‐synaptic parameters such as rise time and decay time were similar between groups (_rise‐time_ *F*_2,22_ = 2.549, *p* = 0.1010, _decay‐time_ *F*_2,21_ = 0.5821, *p* = 0.5675) (Figure 2D–F). To validate that spontaneous activity derived from excitatory post‐synaptic potentials, slices were bath perfused with CNQX (10 μM) and APV (50 μM). CNQX and APV rapidly blocked spontaneous currents, demonstrating that recordings primarily reflect glutamatergic neurotransmission (*t*_5_ = 5.705, *p* = 0.0023) (Figure 2G,H).

To assess if enhanced GABAergic tone could underlie the reduced excitatory neurotransmission, field potential recordings were performed, and the GABAA receptor antagonist bicuculline (20 μM) was bath perfused. Disinhibition induced by bicuculline was not significantly different when comparing the three groups (*F*_2,34_ = 0.5354, *p* = 0.5903), indicating that the GABAergic tone is not increased (Figure 2J). However, stimulus/response curves were significantly depressed in bicuculline‐treated slices from vulnerable rats (*F*_2,32_ = 4.054, *p* = 0.0269; water drinking control vs. vulnerable: *p* = 0.0230) (Figure 2K,L), thereby further supporting reduced excitatory neurotransmission in the BLA of vulnerable rats following long‐term alcohol consumption. Bath perfusion of antagonists targeting ionotropic glutamate receptors robustly inhibited evoked field potentials, validating that recorded currents were associated with excitatory neurotransmission (*F*_1,20_ = 416.5, *p* < 0.0001) (Figure 2M).

### 3.3 Decreased Excitability in BLA Neurons From Vulnerable Rats

**FIGURE 3:**
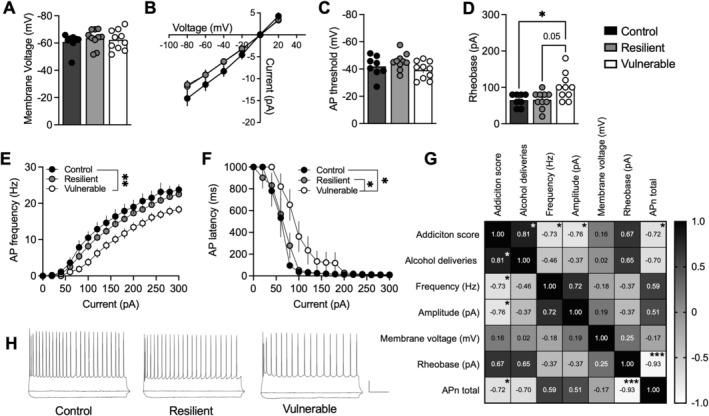
Addiction‐like phenotypic traits are associated withsuppressed intrinsic excitability. (A) Membrane voltage was similar between treatment groups. (B,C) I/V relationship and the threshold for action potential firing were not significantly different when comparing BLA neurons from vulnerable rats with resilient and water drinking controls. (D) BLA neurons from vulnerable rats demonstrated a significant increase in rheobase, suggesting that more current is required to evoke an action potential. (E,F) Reduced action potential frequency and increased latency for action potential firing were observed in BLA neurons from vulnerable rats as compared to the resilient and water control rats. (G) Pearson's correlations demonstrated a significant relationship between behavioural and electrophysiological measures. Addiction score correlated not only with reward deliveries but also with neurotransmission and intrinsic excitability of BLA neurons, suggesting that BLA neurons may be important dictators of AUD‐like behaviour. (H) Representative traces of current‐clamp recordings from all groups. Calibration: 200 ms, 20 mV. Values are presented as mean (±SEM). AP = action potential. Recordings are based on 4–6 animals/treatment group.

To further outline neurophysiological parameters, whole‐cell recordings were conducted in current‐clamp mode. Vulnerable rats demonstrated distinct intrinsic excitability compared to resilient and control rats. Whereas membrane voltage (*F*_2,25_ = 0.3424, *p* = 0.7134) (Figure 3A), current/voltage relationships (*F*_2,25_ = 1.216, *p* = 0.3129) (Figure 3B) and the threshold for action potential firing (*F*_2,25_ = 2.216, *p* = 0.1301) (Figure 3C) were not significantly affected between groups, more current was required to induce action potential firing in BLA neurons from vulnerable rats (*F*_2,25_ = 5.504, *p* = 0.0105) as compared to neurons from resilient rats (*p* = 0.0207) or water drinking controls (*p* = 0.0254) (Figure 3D). Increased rheobase was accompanied by a decreased frequency of action potential firing in response to depolarizing current injections in BLA neurons from vulnerable rats (*F*_2,25_ = 5.843, *p* = 0.0083; vulnerable vs. control *p* = 0.0077; vulnerable vs. resilient *p* = 0.0731), and increased action potential latency (*F*_2,25_ = 4.629, *p* = 0.0195; vulnerable vs. control *p* = 0.0338; vulnerable vs. resilient *p* = 0.0447) (Figure 3E,F,H). Overall, these data indicate a decreased neuronal excitability in the BLA of vulnerable rats, suggesting that synaptic properties in the BLA may be directly associated with AUD‐like behaviour.

### 3.4 Distinct Neurophysiological Properties in BLA Correlate With AUD‐Like Behaviour

To further outline the association between BLA neurotransmission and AUD‐like behaviour, a correlation matrix was implemented to examine the relationship between BLA synaptic activity and behavioural outcomes (Figure 3G). Significant correlations were identified, highlighting the association between behavioural traits and specific synaptic parameters recorded in BLA neurons. The individual addiction scores showed a significant positive correlation with alcohol deliveries (*r* = 0.81, *p* = 0.015), supporting the idea that high alcohol intake is a major determinant influencing alcohol addiction‐like behaviours among outbred rats [5, 18, 19]. Among the electrophysiological measures, both sEPSC frequency (*r* = −0.73, *p* = 0.040) and amplitude (*r* = −0.76, *p* = 0.028) were negatively correlated with the individual addiction score, suggesting that reduced presynaptic input within the BLA and post‐synaptic alterations are associated with a greater predisposition to develop AUD‐like behaviours. Neuronal excitability, as assessed here by the total number of action potentials (APn) fired, also showed a significant negative correlation with addiction scores (*r* = −0.72, *p* = 0.045), with a trend towards an association between rheobase and addiction score (*r* = 0.67, *p* = 0.069). There was further a trend towards an association between action potential firing and the number of alcohol deliveries (*r* = 0.70, *p* = 0.055) and a strong correlation between rheobase and the number of action potentials fired (*r* = −0.93, *p* < 0.001). This relationship suggests that diminished BLA neuronal excitability is associated with increased propensity to express AUD‐like behaviour in rats.

## 4 Discussion

Alcohol use disorder is a multisymptomatic condition characterized by physical, psychological and molecular alterations, some of which can be effectively modelled in laboratory animals. Voluntary long‐term alcohol consumption in rodents has a high predictive validity in humans and is a prerequisite for the development of compulsive‐like drinking behaviours resembling clinical features of addiction [20, 21]. In this study, we identified subpopulations of rats that, following prolonged (> 4 months) alcohol consumption, were categorized as resilient or vulnerable to AUD‐like behaviours and investigated the neurophysiological properties associated with these phenotypes focusing on the BLA. Our findings revealed distinct differences in synaptic activity and intrinsic excitability of neurons in the BLA of rats displaying an AUD‐vulnerable phenotype compared to resilient rats and water drinking controls. The observed properties of BLA neurons further correlated with addiction score and alcohol deliveries, further supporting a role for the BLA in compulsive‐like alcohol seeking and consumption.

To further understand the neurophysiological characteristics of vulnerable and resilient rats, we used ethanol‐naïve rats that were housed in parallel throughout the study period. These animals were primarily included as we speculated that neurotransmission might be distinct not only in vulnerable rats but also in rats that were resilient to AUD‐like behaviour. Furthermore, if neurophysiological characteristics would be present from start and not elicited by repeated ethanol intake and withdrawal periods, recordings from water drinking controls would theoretically demonstrate a higher variance as they should represent both extremes. This was, however, not the case, as synaptic properties were similar when comparing water drinking controls with resilient rats. Considering that the number of ethanol deliveries correlated with addiction score, ethanol intake might have been too low to elicit neurophysiological transformations. However, it is also possible that resilient rats are less susceptible to ethanol‐induced neuroplasticity. It should be noted that ethanol‐naïve rats were never exposed to the operant boxes, and these controls do thus not account for neuroadaptations elicited by instrumental learning. However, previous studies performed in other brain regions have not demonstrated sustained effects on neurotransmission when comparing yoked conditions with saline‐self‐administering animals [22].

Chronic ethanol exposure has previously been shown to induce significant changes in synaptic transmission and neuronal connectivity in the amygdala [23], and a hyperGABAergic state in the central nucleus of the amygdala has been associated with alcohol dependence [24, 25]. However, the impact of long‐term alcohol consumption and the progression to compulsive‐like drinking on glutamatergic synaptic mechanisms in the BLA has not been fully outlined. As disrupted excitatory glutamatergic signalling in the BLA may impair the processing of adaptive emotional and reward signals and skew the motivational landscape towards compulsive‐like alcohol seeking and consumption behaviour, we explored potential alterations in glutamatergic transmission by examining the spontaneous excitatory neurotransmission in the BLA in relation to AUD‐like behaviours. Voltage‐clamp recordings indicated a reduced frequency of sEPSCs recorded in BLA neurons from vulnerable rats as compared to the resilient and control groups. This might suggest an altered control over the BLA from projecting brain regions that modulate synaptic activity and contribute to the overall functional connectivity of the BLA.

In addition to reduced sEPSC frequency, the data presented here demonstrated a concomitant decrease in sEPSC amplitude in vulnerable rats, indicating BLA post‐synaptic transformations associated with the vulnerability to develop AUD‐like behaviours. It should be noted that GABAergic neurotransmission was not inhibited during whole‐cell recordings. Although this was done to avoid pharmacological manipulation of the system, it is possible that a subset of currents recorded is associated with GABAA receptor activation. However, sEPSC recordings performed with internal solution containing K‐gluconate were fully blocked by a cocktail of AMPA and NMDA antagonists, which is in agreement with findings in other brain regions [22]. Thus, recorded currents should primarily reflect activation of glutamatergic currents. Furthermore, CNQX and APV further blocked evoked field potentials, further demonstrating that excitatory neurotransmission is assessed in these recordings [26]. Field potentials demonstrated a similar disinhibition induced by GABAA receptor antagonist independent of group category, suggesting that group differences are not driven by changes in GABAergic tone. In addition, fEPSP amplitudes, recorded during conditions where GABAA receptors were fully blocked, were selectively depressed in brain slices from vulnerable rats, thereby further supporting a hypoglutamatergic state. Although AMPA receptor subunit 2 has been demonstrated to be downregulated in males diagnosed with AUD [27] and thus might contribute to the decrease in amplitude, further studies are required to outline neurobiological transformations underlying these findings. Importantly, sEPSC amplitude in individual animals correlated with the addiction score, further supporting a role for BLA neurotransmission in AUD‐like behaviour.

Although the data presented here revealed reduced excitability in BLA neurons from rats displaying an AUD‐like behaviour, other studies have reported increased excitability of the BLA following chronic alcohol exposure. However, many of these studies incorporate preclinical models with a stress component, which may contribute to the observed hyperexcitability [28]. For instance, animal models involving chronic intermittent exposure (CIE) vapour inhalation [29], exposure to early‐life stress [30] or withdrawal periods [31] often report heightened BLA excitability, suggesting that the observed differences may reflect the distinct contributions of stress to the neural adaptations in the BLA. Especially, studies using the CIE model consistently report increased glutamatergic presynaptic function within the BLA [32, 33], but the paradigm may induce elevated anxiety‐like behaviour during withdrawal [34]. This could further exacerbate BLA glutamatergic function, similar to the enhancements observed in animals subjected to stress, anxiety or fear‐conditioning procedures [35, 36, 37, 38, 39]. In fact, changes in the regulation of BLA excitability have previously been demonstrated to underlie pathological anxiety, fear‐related disorders and maladaptive behaviours associated with drug addiction [28, 40]. Contrasting findings may also be linked to the distinct alcohol administration paradigms themselves. Active and passive alcohol administration have been shown to recruit different neuronal ensembles, which could contribute to the observed variations in glutamatergic transmission [41]. Moreover, the alterations in BLA neuronal excitability during chronic alcohol exposure may exhibit dynamic remodelling, evolving throughout different stages of alcohol dependence. Notably, for the data presented here, recordings were conducted in direct vicinity to a self‐administration session, thus not during withdrawal periods. Interestingly, temporal adaptations in the excitability of BLA neurons projecting to the dorsomedial striatum occur during withdrawal after CIE, with action potential firing being significantly reduced 3‐day post‐withdrawal, closer to our recording timeframe, but markedly increased at 1‐ and 2‐week post‐withdrawal [32]. This suggests that BLA neurons may undergo distinct, time‐dependent shifts with regard to excitability following the cessation of alcohol exposure. Lastly, the experimental layout employed here assessed neurotransmission in rats that had gained access to alcohol for over 4 months. Thus, the extensiveness of the protocol employed, as well as the age of the animals, could have influenced the experimental outcome [42].

Both sEPSC frequency, amplitude and intrinsic excitability were suppressed in BLA neurons of rats displaying an AUD‐vulnerable phenotype, but the current study does not distinguish between projection‐specific inputs or outputs, limiting the ability to determine whether the observed effects are pathway specific. Based on previous studies, glutamatergic projections from the prelimbic cortex, which are prevalent in the BLA [43], may be especially relevant considering the presumed role of this brain structure in AUD‐like behaviour [44, 45]. Considering the reciprocal connection between BLA and prelimbic cortex, the reduced excitability of BLA neurons observed in this study may further contribute to the decreased presynaptic activity observed in the prelimbic cortex of AUD‐like vulnerable rats [5]. Putatively, reduced activity of glutamatergic projections from the BLA to upstream targets, such as the prelimbic cortex, may diminish cognitive control over alcohol‐seeking behaviours and play a key role in the development of an AUD‐like addiction phenotype.

Although the data presented here demonstrated altered synaptic transmission in the BLA of vulnerable rats, one limitation of our study is that we could not determine whether vulnerable individuals already exhibited an altered BLA neurobiological profile prior to alcohol exposure, potentially indicating a physiological vulnerability to developing AUD‐like behaviours, or if neuroadaptations in the BLA occurred in response to protracted alcohol intake. In fact, the reduced neuronal activity in the BLA may impair not only the processing of reward stimuli [46] but also the regulation of stress and anxiety [47], leading to altered behavioural responses that are commonly seen in AUD. We also acknowledge that the generalizability of our findings is limited by the exclusive use of male rats, as sex differences in BLA functionality and alcohol‐related behaviours are well documented [29, 48, 49]. Future studies incorporating both sexes will be essential to determine whether similar neuroadaptations occur in females and to explore potential sex‐specific mechanisms underlying AUD. Lastly, in this study, we focus on the extremes of the population, generating a low number of animals in each group and thereby a risk of an underpowered study.

In conclusion, our findings collectively suggest that AUD‐like behaviour, assessed after prolonged alcohol consumption, is associated with a distinct neurophysiological profile in the BLA that involves reduced activity of excitatory inputs, reduced post‐synaptic activation and suppressed intrinsic excitability. These results underscore a potential link between neurobiology and behavioural features associated with AUD. Such insights are pivotal for advancing the development of targeted pharmacological interventions to treat AUD in humans.

## Author Contributions

Ana Domi and Louise Adermark designed the experiments. Ana Domi carried out the behavioural experiments. Davide Cadeddu and Erika Lucente carried out the electrophysiological experiments. Ana Domi, Louise Adermark, Davide Cadeddu and Erika Lucente performed the data analysis. Ana Domi, Davide Cadeddu and Louise Adermark wrote and revised the manuscript. Mia Ericson and Bo Söderpalm provided critical comments, assisted with data interpretation and revised the manuscript. All authors reviewed and approved the manuscript. Artificial intelligence tools or technologies were not used in the writing of the manuscript.

## Conflicts of Interest

The authors declare no conflicts of interest.

## Data Availability

The data that support the findings of this study are available on request from the corresponding author. The data are not publicly available due to privacy or ethical restrictions.
